# Enhanced Bioactivity of Micropatterned Hydroxyapatite Embedded Poly(L-lactic) Acid for a Load-Bearing Implant

**DOI:** 10.3390/polym12102390

**Published:** 2020-10-17

**Authors:** Sae-Mi Kim, In-Gu Kang, Kwang-Hee Cheon, Tae-Sik Jang, Hyoun-Ee Kim, Hyun-Do Jung, Min-Ho Kang

**Affiliations:** 1 Department of Materials Science Engineering, Seoul National University, Seoul 08826, Korea; bluelear@snu.ac.kr (S.-M.K.); haeronggu@snu.ac.kr (I.-G.K.); adonis9787@snu.ac.kr (K.-H.C.); kimhe@snu.ac.kr (H.-E.K.); 2 Department of Materials Science and Engineering, Chosun University, Gwangju 61452, Korea; tsjang@chosun.ac.kr; 3 Department of Biomedical-Chemical Engineering, Catholic University of Korea, Bucheon-si 03083, Korea; 4 Department of Chemical and Biological Engineering, Seoul National University, Seoul 08826, Korea

**Keywords:** poly(L-Lactic) acid, hydroxyapatite, patterning, load-bearing implant, biocompatibility

## Abstract

Poly(L-lactic) acid (PLLA) is among the most promising polymers for bone fixation, repair, and tissue engineering due to its biodegradability and relatively good mechanical strength. Despite these beneficial characteristics, its poor bioactivity often requires incorporation of bioactive ceramic materials. A bioresorbable composite made of PLLA and hydroxyapatite (HA) may improve biocompatibility but typically causes deterioration in mechanical properties, and bioactive coatings inevitably carry a risk of coating delamination. Therefore, in this study, we embedded micropatterned HA on the surface of PLLA to improve bioactivity while eliminating the risk of HA delamination. An HA pattern was successfully embedded in a PLLA matrix without degeneration of the matrix’s mechanical properties, thanks to a transfer technique involving conversion of Mg to HA. Furthermore, patterned HA/PLLA’s biological response outperformed that of pure PLLA. These results confirm patterned HA/PLLA as a candidate for wide acceptance in biodegradable load-bearing implant applications.

## 1. Introduction

Biodegradable polymers that can be resorbed in the body have been intensively investigated given their elimination of the need for a second surgery after the function of the implant has been fulfilled [1,2]. Among biodegradable polymers, poly(L-lactic) acid (PLLA) is considered among the most desirable for fracture fixation, repair, and tissue engineering due to its relatively high mechanical strength [3,4,5]. However, its degradation byproducts create an acidic environment that causes inflammatory responses [6,7,8]. Moreover, its poor bioactivity [9,10] often requires incorporation of bioactive ceramic, restricting the active clinical use of PLLA [11].

To date, composites made of PLLA and bioactive ceramics such as hydroxyapatite (HA) have been extensively developed to improve clinical performance [12,13]. The incorporation of HA particles into the PLLA matrix not only neutralizes the degraded acidic monomers, but also increases tissue compatibility due to the compositional similarity between HA and hard tissue [14,15]. However, the interface between the PLLA matrix and HA particles often weakens the overall strength of the composite owing to their low mutual affinity, thus limiting this combination’s application in load-bearing areas [16,17]. Surface coatings constitute another approach for increasing bioactivity without altering the mechanical characteristics of the PLLA matrix [18]. However, for surface coatings a critical problem is the risk of delamination, which results from the low bonding strength between the PLLA substrate and the coating layer. Furthermore, the application of a ceramic coating layer on a polymer matrix has been rarely reported because ceramic coating layers tend to be less flexible than their polymer matrices [19].

Therefore, the objective of the present work is to propose an alternative approach for increasing the surface bioactivity of PLLA without the concerns of PLLA matrix weakening or HA coating layer delamination. This alternative approach embeds an HA micropattern on the surface of the PLLA matrix. A large amount of HA micropattern is exposed on the surface in order to prompt a bioactivity increase, which is difficult to achieve through simple powder blending. The microstructure, mechanical performance, and in vitro biocompatibility of patterned HA/PLLA are evaluated.

## 2. Materials and Methods

### 2.1. Preparation of Pure PLLA and PLLA/HA Composite

Pure PLLA granules (P9001, Pure Eco, Paju, Korea) were put in a rigid die with a diameter of 12 mm and compressed at 190 °C under a uniaxial load of ~22 MPa for 10 min. For the PLLA/HA composite, a PLLA/HA mixture containing 20 vol% of HA was prepared by mechanically blending PLLA and HA particles via shear mixing at 190 °C for 30 min. The resultant PLLA/HA mixture followed the same procedure as applied to pure PLLA rod fabrication.

### 2.2. Preparation of Patterned HA/PLLA

**Figure 1:**
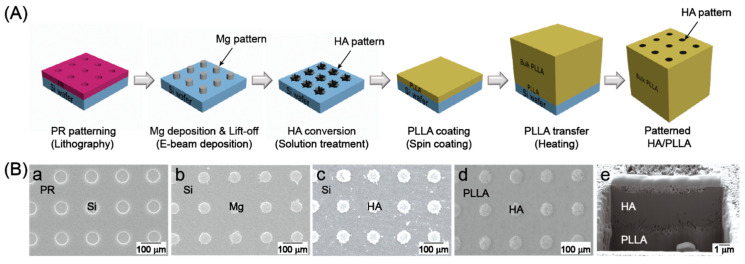
(**A**) Scheme of the fabrication process of patterned hydroxyapatite (HA)/Poly(L-lactic) acid (PLLA) for load-bearing application. (**B**) Scanning electron microscopy (SEM) images of (a) Photo resist (PR) patterning, (b) Mg deposition and lift-off, (c) HA conversion, (d) PLLA transfer, and (e) cross-section of patterned HA/PLLA.

Using a photoaligner (Karl-Suss MA-6 II), positive photoresist (AZ5214) was patterned with hole arrays with 10-μm-diameter holes and 20 μm between hole centers on a silicon wafer. After the development of the photoresist pattern, Mg deposition proceeded by e-beam evaporator (EVACO-EB800R, DR Vacuum Inc., Seoul, Korea) on the photoresist-patterned surface with a thickness of 1 μm. Then, the photoresist layer was lifted off in acetone to leave only the Mg pattern on the wafer. This Mg array was converted to HA through solution treatment as described in a previous study [20]. Briefly, the samples were treated in a 0.05 M ethylenediaminetetraacetic acid calcium disodium salt hydrate (Ca-EDTA)/0.05 M potassium dihydrogen phosphate (KH_2_PO_4_) aqueous solution. During the treatment, pH was adjusted to 8.9 using a sodium hydroxide (NaOH) solution, and the temperature of the solution was kept at 90 °C for 2 h. Next, 5 wt% PLLA in dichloromethane (DCM) was spin-coated on the HA-patterned wafer and dried in a 37 °C oven overnight. For the final step, the HA/PLLA pattern on the wafer was transferred to a pure PLLA rod by melting the interface between pattern and rod on a 250 °C hot plate. The fabrication process scheme and surface morphology of the patterned HA/PLLA are shown in Figure 1.

### 2.3. Microstructure and Surface Chemical Behaviors

Scanning electron microscopy (SEM, JSM-5600; JEOL, Tokyo, Japan) was used to observe the surface morphologies of the pure PLLA, HA/PLLA, and patterned HA/PLLA specimens. The cross-sectional morphology of the patterned sample was observed by field emission scanning electron microscopy (FESEM; AURIGA, Carl Zeiss, Oberkochen, Germany) equipped with a focused ion beam. To confirm the HA conversion after solution treatment, the crystalline structure of the patterned HA/PLLA was examined by X-ray diffraction (XRD; D8-Advance, Bruker Co., Karlsruhe, Germany). Contact angle tests were performed to investigate the wettability and hydrophobicity of the prepared samples. Static water contact angles were measured using the sessile drop method on a goniometer (PHX300, S.E.O., Suwon, Korea) at ambient temperature. A water droplet of 15-μL volume was used in each measurement. Seven measurements were conducted on different locations for each sample.

### 2.4. Mechanical Behaviors

To evaluate the mechanical properties of pure PLLA, HA/PLLA, and patterned HA/PLLA, tension tests were conducted in accordance with ASTM 638 [21,22]. Specimens with a thickness of 1.5 mm and a gauge length of 8 mm were elongated uniaxially at a constant crosshead speed of 1 mm/min. The stress and strain responses of the specimens were monitored throughout the tensile strength tests. Five specimens were tested.

### 2.5. In Vitro Biological Behaviors

For the in vitro cell tests, MC3T3-E1 (ATCC, CRL-2593), was used to assess the cellular response to the specimen surface. The pre-incubated cells were seeded on specimens at a density of 5.0 × 103 or 2.5 × 103 cells/cm^2^ to evaluate cell attachment or proliferation, respectively.

After culturing for 24 h, the attached cells were observed by SEM (JSM-5600; JEOL, Japan). Prior to the SEM observations, the samples were fixed with 2.5% glutaraldehyde for 10 min, dehydrated in graded ethanol (70, 95, and 100% ethanol in sequence), and immersed in hexamethyldisilazane for 10 min.

Cell proliferation was determined after 3 and 5 d of culturing using a Cyquant cell proliferation assay kit (C7026, Invitrogen Corp., Carlsbad, CA, USA). Before measurements, the cells that adhered to the samples were detached and suspended in a fluorescent dye solution. The DNA level of the detached cells was measured using a multiple plate reader (Victor3, PerkinElmer, Boston, MA, USA) at wavelengths of 480/535 nm. The measured fluorescence values were converted to the DNA content using a DNA standard curve.

### 2.6. Statistical Analysis

Mechanical and biological experiments were performed on five specimens, and the experimental results were expressed as the mean value ± standard deviation. The normality of variables was tested using the Student’s t-test. Differences among the three groups (pure PLLA, PLLA/HA, and patterned HA/PLLA) were determined using a one-way analysis of variance followed by Tukey’s post hoc comparison test, with *p* < 0.05 considered a statistically significant difference.

## 3. Results and Discussion

### 3.1. Microstructure and Surface Chemical Properties

**Figure 2:**
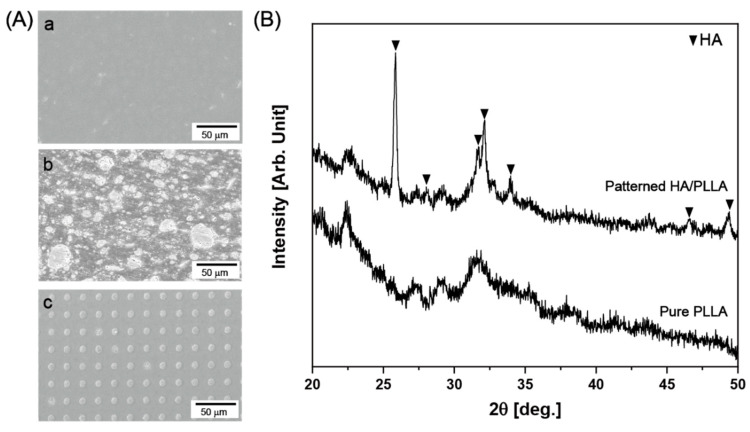
(**A**) SEM images of (a) pure PLLA, (b) HA/PLLA, and (c) patterned HA/PLLA. (**B**) XRD spectra of pure PLLA and patterned HA/PLLA.

Figure 2A presents the surface morphology of pure PLLA, HA/PLLA, and patterned HA/PLLA. HA microdots with a diameter of ~10 μm were uniformly embedded at an interspacing distance of ~10 μm on the PLLA surface and were well embedded in the PLLA matrix. HA particle was also well dispersed in the PLLA, with particle size varying from 1 to more than 50 μm, implying that the agglomeration of HA particle was unavoidable. There were no noticeable large defects, such as voids or cracks, under any condition, suggesting that compression molding at 190 °C performed well in sample production. The surface crystalline structures of pure PLLA and patterned HA/PLLA were analyzed by XRD, as shown in Figure 2B. Compared with that of pure PLLA, the XRD pattern of patterned HA/PLLA exhibited clear peaks originating from HA, showing that solution treatment fully converted the Mg micropattern to an HA micropattern.

### 3.2. Wettability and Mechanical Properties

A contact angle test was performed to evaluate the hydrophilicity of each prepared sample. Since the specimen surface was flat in all conditions due to the high-temperature pattern transfer method, it can be assumed that the hydrophilicity of the specimens was affected only by the chemical composition of the surface. As reported in many studies, pure PLLA showed hydrophobic properties, displaying a contact angle of about 80° with static water [23,24,25]. In the case of HA/PLLA, a slightly smaller wetting angle of around 74° was observed, even though hydrophilic HA particle was dispersed over the surface. On the other hand, a dramatically lower contact angle of around 20° was detected for patterned HA/PLLA. Notably, the exposed HA area in both conditions was fixed at 20%, implying that the periodic arrangement of HA particles made a significant impact on the increased hydrophilicity of the specimens.

**Figure 3:**
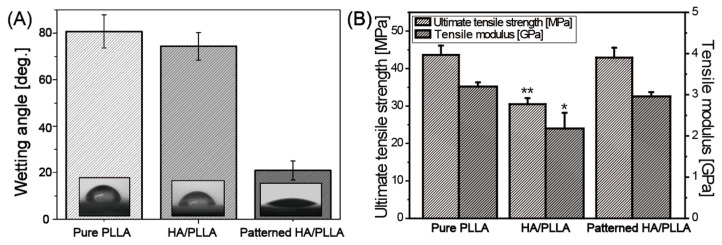
(**A**) Wetting angle and (**B**) tensile properties of pure PLLA, HA/PLLA, and patterned HA/PLLA (Statistically significant vs. pure PLLA and patterned HA/PLLA: * *p* < 0.05, ** *p* < 0.01).

In order to further evaluate the effect of HA patterning on mechanical characteristics, the mechanical properties of specimens were examined via tensile strength tests. Figure 3B presents maximum tensile strength and elastic modulus results for pure PLLA, HA/PLLA, and patterned HA/PLLA specimens. Since the ceramic HA particle was mixed into the PLLA matrix, the tensile strength and modulus were both significantly lower (tensile strength 30.5 MPa, tensile modulus 2.2 GPa) than for pure PLLA (tensile strength 43.7 MPa, elastic modulus 3.2 GPa) because of the low affinity between HA particle and PLLA matrix, as discussed above. In the case of patterned HA/PLLA, the HA pattern rarely affected the mechanical properties of the bulk PLLA matrix, thus there were no significant differences in both the tensile strength and modulus between pure PLLA and patterned HA/PLLA. The tensile properties of each specimen are summarized in Table 1. Generally, as the amounts of bare HA fillers increase, tensile properties of the composites decrease, owing to poor dispersion of HA within the PLA matrix [26,27]. The HA content in the HA/PLLA sample was 20 vol%, and this value was selected based on the calculation of the HA content on the surface of the patterned HA/PLLA. However, the 20 vol% of HA in HA/PLLA sample deteriorated the mechanical properties, which showed similar results to those reported in previous studies [26,27]. It should be noted that patterned HA/PLLA maintained tensile properties and did not show a significant difference when compared with those of pure PLLA, and these values compare favorably with the tensile strength (−50 MPa) and stiffness (3–20 GPa) of natural cortical bone in transverse loading [28].

### 3.3. In Vitro Cell Responses

**Figure 4:**
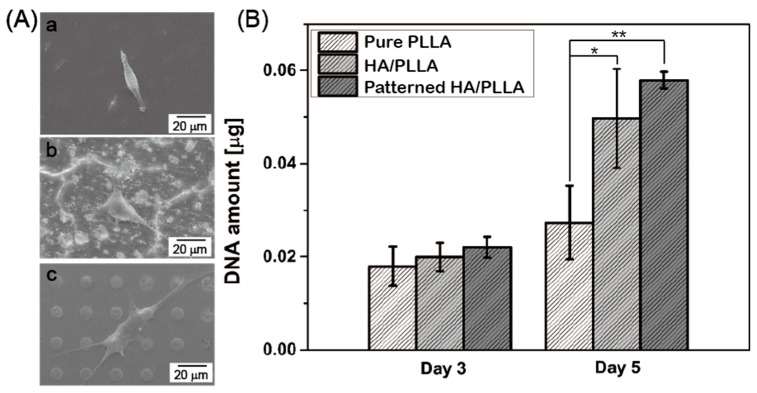
(**A**) SEM images of cell morphology after 1 d of culturing of (a) pure PLLA, (b) HA/PLLA, and (c) patterned HA/PLLA. (**B**) DNA levels of MC3T3-E1 cells after 3 and 5 d of culturing on pure PLLA, HA/PLLA, and patterned HA/PLLA (* *p* < 0.05, ** *p* < 0.01).

In vitro biocompatibility of pure PLLA, HA/PLLA, and patterned HA/PLLA was examined by observing the morphology of the initial cell attachment and cell proliferation. Figure 4A shows representative SEM images of specimens after 1 d of cell culturing. Generally, none of the samples showed any signs of cytotoxicity. However, cells on pure PLLA adhered poorly with elongated morphology in the restricted direction. In contrast, cells on the surfaces of HA/PLLA and patterned HA/PLLA showed filopodia extensions and flattened morphology. Bioactive HA acted as attractive sites for cells to easily adhere and improved cell affinity on the PLLA surface [29,30]. Furthermore, the degree of cell proliferation on the pure PLLA, HA/PLLA, and patterned HA/PLLA after 3 d and after and 5 d of cell culturing was examined using a DNA assay, as shown in Figure 4B. The specimens with an HA component showed significantly larger absorbance values (*p* < 0.05) than those of pure PLLA specimens after 5 d of culturing. These findings suggest that the HA-containing treatments significantly facilitate the attachment and proliferation of MC3T3-E1 cells, which would be expected to provide enhanced osseointegration ability in vivo and promote fast recovery for patients. The patterned HA/PLLA specimens’ level of proliferation was similar to that obtained for HA/PLLA, due to equal amounts of HA on the surface of each. However, patterned HA/PLLA has greater potential HA incorporation capacity, rather than particle aggregation as seen in HA/PLLA.

## 4. Conclusions

Patterned HA/PLLA was successfully developed through photo-lithography in conjunction with Mg deposition and HA conversion on a patterned wafer. Patterning was applied only on the surface, so it enabled the modification of surface-dependent properties, such as cell attachment and proliferation, while matrix-dependent properties such as mechanical strength and elastic modulus were maintained. Patterned HA/PLLA exhibited better cellular responses than pure PLLA, implying cell affinity could be effectively modulated by HA patterning. In addition, the mechanical properties of patterned HA/PLLA, including tensile strength and elastic modulus, were retained and matched those of natural cortical bone. In contrast, PLLA/HA showed a significant deterioration in mechanical properties, performing distinctively poorly in both tensile strength and elastic modulus. These findings indicate that HA patterns could significantly modulate in vitro biocompatibility of PLLA without disturbing the polymer’s intrinsic mechanical properties. With the help of this favorable combination of properties, patterned HA/PLLA could potentially be used for biodegradable load-bearing implants.

## Figures and Tables

**Table 1:** Tensile properties of PLLA and its composites.

	Pure PLLA	HA/PLLA	Patterned HA/PLLA
Ultimate tensile strength [MPa]	43.7 ± 2.5	30.5 ± 1.6	42.9 ± 2.7
Elastic modulus [GPa]	3.4 ± 0.1	2.2 ± 0.4	3.0 ± 0.1
